# Multiple Coronary Artery Aneurysms and Coronary–Pulmonary Artery Fistula Associated with Vieussens’ Arterial Ring

**DOI:** 10.70352/scrj.cr.25-0731

**Published:** 2026-02-27

**Authors:** Akito Kuwano, Masaru Yoshikai, Satoshi Ohtsubo, Kiyokazu Koga, Nozomi Yoshida, Naoyo Nishida

**Affiliations:** 1 Department of Cardiovascular Surgery, Shin-Koga Hospital, Kurume, Fukuoka, Japan; 2 Department of Pathology, Shin-Koga Hospital, Kurume, Fukuoka, Japan

**Keywords:** Vieussens’ arterial ring, coronary artery aneurysm, coronary–pulmonary artery fistula

## Abstract

**INTRODUCTION:**

Vieussens’ arterial ring (VAR) is a vascular anomaly characterized by a communication between the conus branch of the right coronary artery (or the isolated conus artery) and the left anterior descending artery. Pathological changes of VAR, including dilation or aneurysmal formation, are exceedingly rare, and the mechanisms underlying these changes have yet to be elucidated.

**CASE PRESENTATION:**

A 65-year-old woman presented with dyspnea. Contrast-enhanced CT revealed that the isolated conus artery, arising directly from the right sinus of Valsalva, communicated with the left anterior descending artery, corresponding to VAR. The vessel exhibited beaded dilation and aneurysmal formation, and formed a fistula to the main pulmonary artery. We performed surgical resection of the coronary artery aneurysms and closure of the fistulous connection. The postoperative course was uneventful. Histopathological examination of the resected coronary aneurysm wall revealed thinning of the vessel wall and disruption of elastic fibers in the media. Of particular note was the prominent infiltration of CD8-positive T cells in the VAR specimen, in addition to the presence of atherosclerotic changes.

**CONCLUSIONS:**

We present a rare case in which VAR developed multiple coronary artery aneurysms and a coronary - pulmonary artery fistula, and discuss the potential mechanisms underlying its pathological alterations. Our findings suggest that inflammation associated with CD8-positive T cells may contribute to vascular wall fragility in VAR.

## Abbreviations

CAA
: coronary artery aneurysm
CPAF
: coronary–pulmonary artery fistula
LAD
: left anterior descending artery
RCA
: right coronary artery
VAR
: Vieussens’ arterial ring

## INTRODUCTION

VAR is a vascular anomaly in which the conus branch of the RCA, or the isolated conus artery,^1)^ communicates with the LAD.^2)^ VAR is infrequently identified on imaging, and cases in which VAR is associated with pathological changes, including aneurysmal formation, are exceedingly rare.^3)^ We present a rare case in which VAR was associated with multiple CAAs and a CPAF, and discuss the potential mechanisms underlying its pathological alterations based on histopathological findings.

## CASE PRESENTATION

**Fig. 1:**
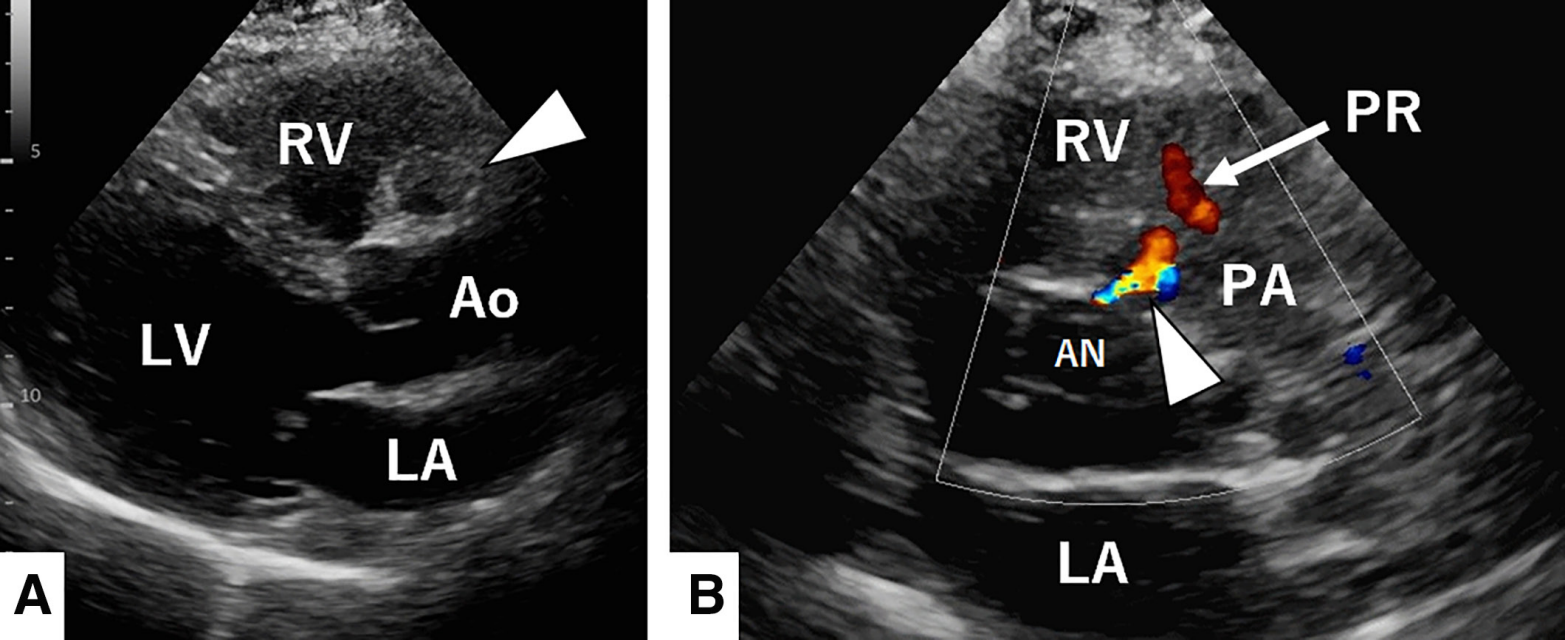
Transthoracic echocardiographic findings. Transthoracic echocardiography reveals a round structure measuring 19 × 13 mm near the right sinus of Valsalva (arrowhead) (**A**: long-axis view) and also demonstrates diastolic flow entering the main pulmonary artery immediately above the pulmonary valve (arrowhead) (**B**: short-axis view). AN, aneurysm; Ao, aorta; LA, left atrium; LV, left ventricle; PA, pulmonary artery; PR, pulmonary valve regurgitation; RV, right ventricle

**Fig. 2:**
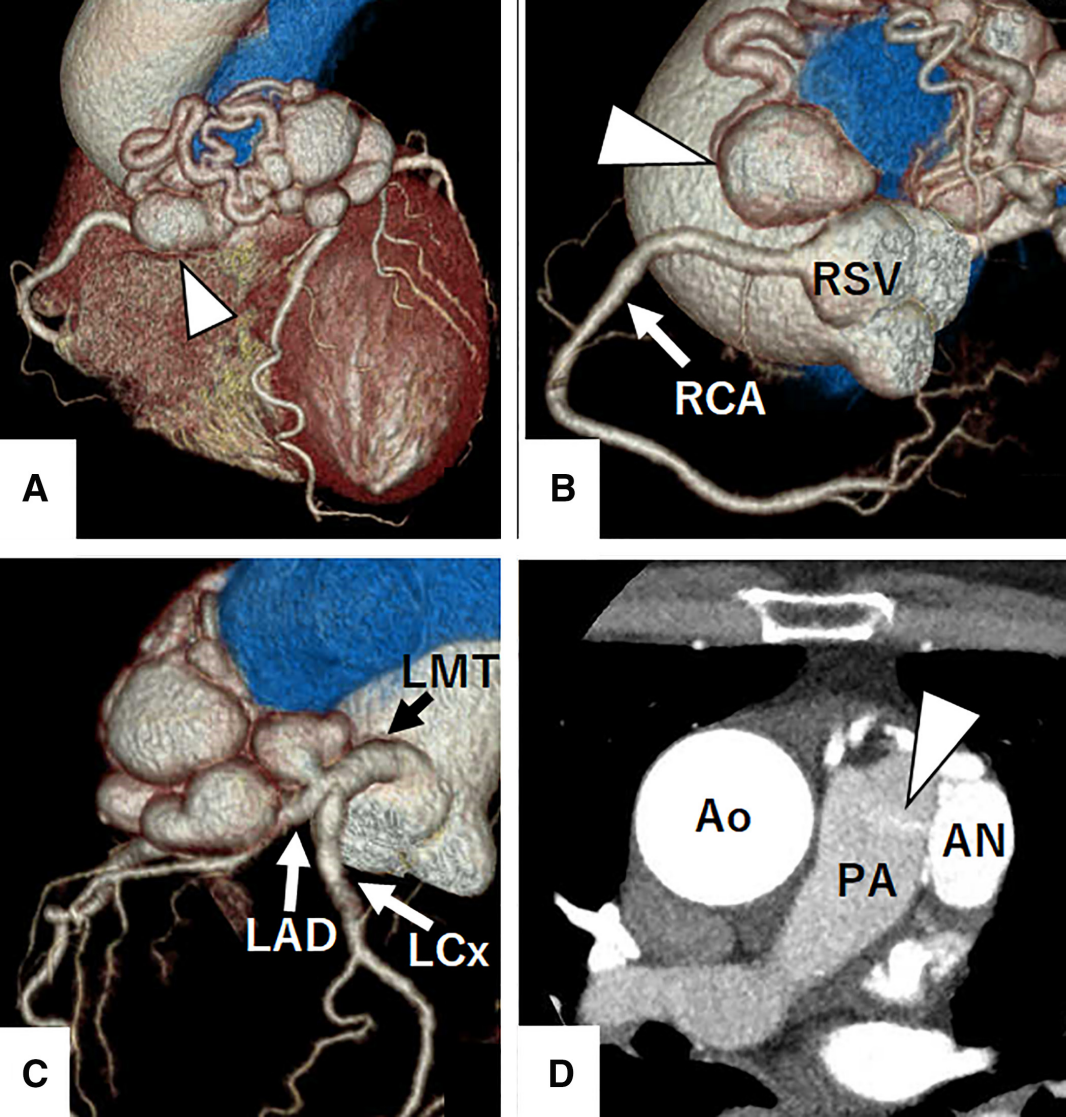
Preoperative contrast-enhanced CT findings. Contrast-enhanced CT reveals an aneurysm of the isolated conus artery, which arises directly from the right sinus of Valsalva (arrowheads in **A**, **B**). The vessel subsequently shows a beaded appearance with segmental dilatations and aneurysmal changes, and communicates with the proximal left anterior descending artery (**C**), forming Vieussens’ arterial ring. In the early phase of contrast enhancement, opacification of the main pulmonary artery is observed, indicating the presence of a coronary-to-pulmonary artery fistula (arrowhead) (**D**). AN, aneurysm; Ao, aorta; LAD, left anterior descending artery; LCx, left circumflex artery; LMT, left main trunk; PA, pulmonary artery; RCA, right coronary artery; RSV, right sinus of Valsalva

**Fig. 3:**
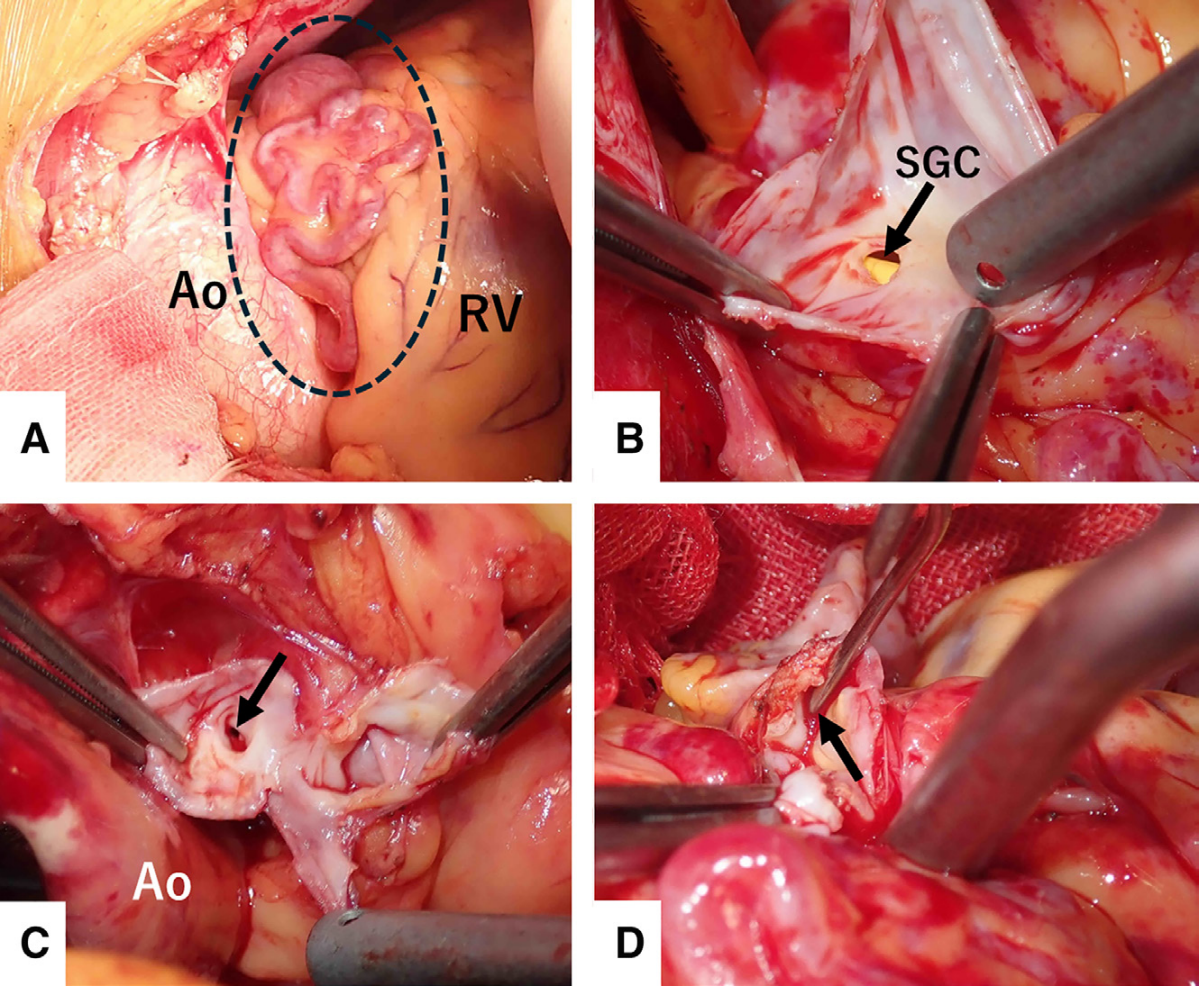
Operative findings. Vieussens’ arterial ring is visualized, with the vessel showing a beaded appearance, segmental dilatations, and marked tortuosity (dotted circle) (**A**). The opened coronary aneurysm located adjacent to the pulmonary artery shows a fistulous connection to the pulmonary artery, through which part of a Swan–Ganz catheter is visible (**B**). The inflow of the aneurysm arising from the isolated conus artery is shown (arrow) (**C**). The inflow of the aneurysm originating from the LAD is visualized (arrow) (**D**). Ao, aorta; LAD, left anterior descending artery; RV, right ventricle; SGC, Swan–Ganz catheter

**Fig. 4:**
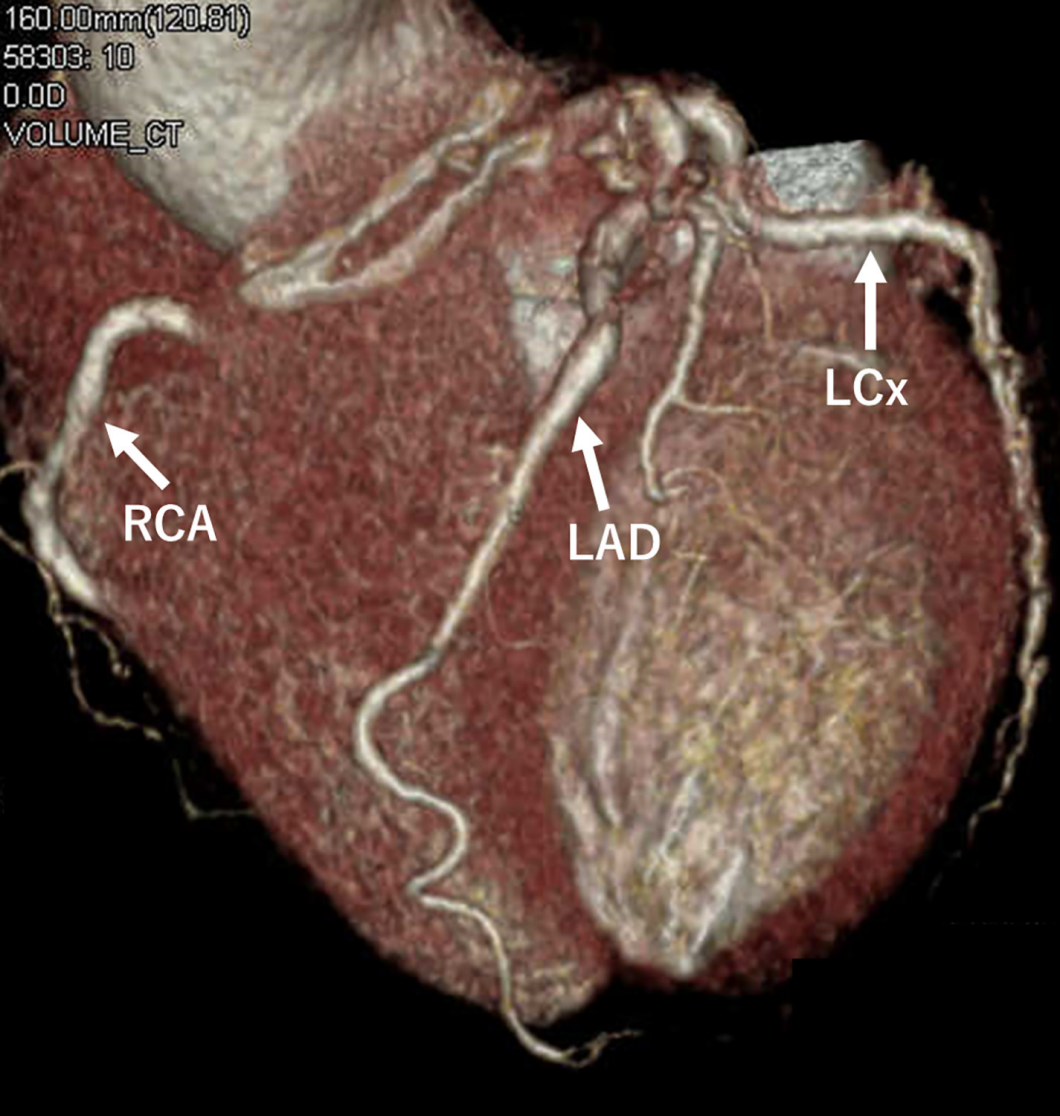
Postoperative contrast-enhanced CT findings. Postoperative contrast-enhanced CT reveals no residual coronary artery aneurysms. LAD, left anterior descending artery; LCx, left circumflex artery; RCA, right coronary artery

**Fig. 5:**
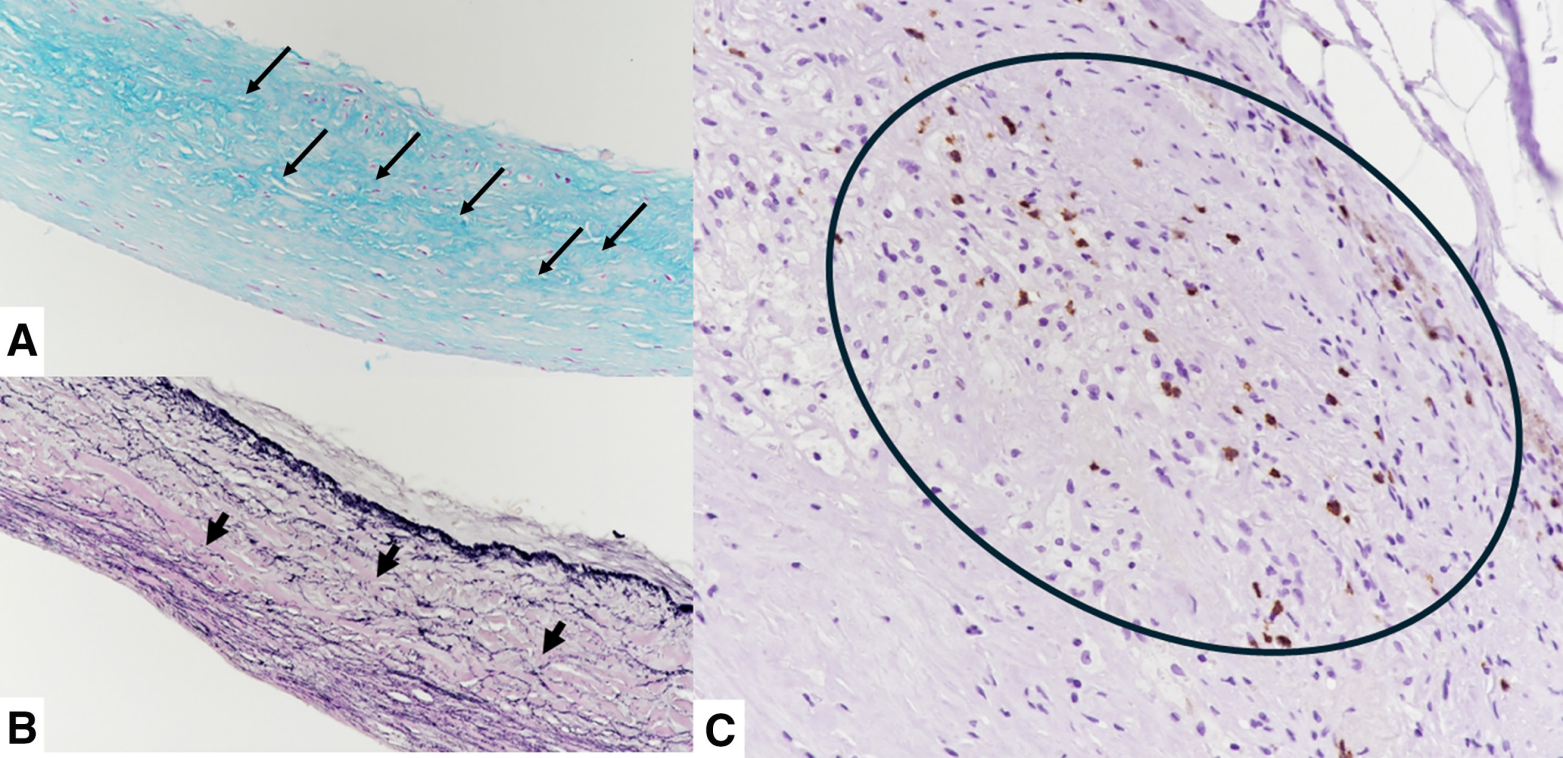
Histopathological findings. Histopathological examination of the resected coronary aneurysm wall reveals degeneration of the tunica media with mucopolysaccharide deposition, which appears as dark blue areas (arrows) (**A**: Aldehyde Fuchsin staining, original magnification ×200), and disruption of elastic fibers (arrows) (**B**: Elastica van Gieson staining, original magnification ×200). Resected VAR shows infiltration of CD8-positive T cells (encircled area, stained in brown) (**C**; immunohistochemical staining for CD8, original magnification ×200). VAR, Vieussens’ arterial ring

A 65-year-old woman with hypertension presented to our hospital with dyspnea. At presentation, she had no chest symptoms or fever, and cardiac auscultation revealed no murmurs. Laboratory tests revealed a normal white blood cell count and a negative C-reactive protein level. Electrocardiography showed a sinus rhythm with no significant ST-T changes. Chest radiograph showed neither cardiomegaly nor pulmonary congestion. Transthoracic echocardiography revealed a round structure measuring 19 × 13 mm near the right sinus of Valsalva and also demonstrated diastolic flow entering the main pulmonary artery immediately above the pulmonary valve (**Fig. 1**). Contrast-enhanced CT revealed a 20 × 20 mm aneurysm of the conus artery, which arose directly from the right sinus of Valsalva (**Fig. 2A**, **2B**). The vessel subsequently showed a beaded appearance with segmental dilatations and aneurysmal changes (maximum, 27 × 19 mm), and communicated with the proximal LAD (**Fig. 2C**). In the early phase of contrast enhancement, opacification of the main pulmonary artery was observed, indicating the presence of a CPAF (**Fig. 2D**). Right heart catheterization revealed no evidence of pulmonary hypertension, and the oximetry run demonstrated a pulmonary-to-systemic ratio of 1:16. Surgical intervention, intended to prevent rupture of the CAAs, was performed via a median sternotomy under cardioplegic arrest (**Fig. 3A**). Upon incision of the CAA adjacent to the pulmonary artery, a fistulous connection to the pulmonary artery was identified, through which the Swan–Ganz catheter was visible (**Fig. 3B**). The fistula was closed with direct sutures. The inflow of the aneurysm arising from the isolated conus artery (**Fig. 2A**, **2B**) was closed with direct sutures (**Fig. 3C**). Similarly, the inflow of the aneurysm originating from the LAD (**Fig. 2C**) was closed in the same manner (**Fig. 3D**). The abnormally beaded, dilated, and tortuous vessels were each ligated and sutured. The postoperative course was uneventful, and postoperative contrast-enhanced CT revealed no residual CAAs or shunts (**Fig. 4**). Histopathological examination of the resected coronary aneurysm wall revealed thinning of the vessel wall, degeneration of the tunica media with mucopolysaccharide deposition, and disruption of elastic fibers (**Fig. 5A**, **5B**). Of particular note was the prominent infiltration of CD8-positive T cells in the VAR specimen, whereas only a small number of CD4-positive T cells were observed, yielding an approximate CD4/CD8 ratio of 1:2, along with mild atherosclerotic changes (**Fig. 5C**). Immunohistochemical staining for CD68 and IgG4 revealed no diagnostically significant findings.

## DISCUSSION

VAR, first described by Raymond de Vieussens in 1706, is a vascular anomaly characterized by a communication between the conus branch of the RCA or the isolated conus artery and the LAD.^1,2)^ This anomaly is rare, having been identified in only 11 of 3443 patients (0.0319%) who underwent coronary CT angiography in the series reported by Doğan et al.^3)^ Embryologically, VAR represents a remnant of the conotruncal ring and is usually asymptomatic; however, in patients with ischemic heart disease, it develops as a collateral pathway and has important clinical implications.^4)^ Moreover, the potential role of VAR in percutaneous coronary intervention for chronic total occlusion has been highlighted;^5)^ therefore, understanding its anatomical characteristics and clinical implications is essential for clinicians engaged in coronary interventions. According to the classification of VAR proposed by Christodoulo et al.,^5)^ the most common variant involves the conus branch of the RCA communicating the proximal LAD, accounting for 51.8% of cases. By contrast, the variant in which the isolated conus artery arising from the right sinus of Valsalva communicates with the proximal LAD, as in the present case, was reported in 10 of 56 patients (17.9%).

CAA and CPAF are rare coronary anomalies. The reported prevalence of CAA ranges from 0.3% to 5.3%, whereas giant CAAs (>20 mm) occur in only approximately 0.02% of cases and are associated with an increased risk of rupture.^6,7)^ By contrast, CPAF has been reported to occur in 0.17%–0.68% of the population.^8)^ Given the rarity of each condition, the coexistence of VAR with both CAA and CPAF is considered extremely uncommon. In recent years, several cases of VAR complicated by CAA and CPAF—similar to the present case—have been reported^9–12)^; however, the pathogenesis of VAR has yet to be fully elucidated. VAR cases complicated by CAA are frequently accompanied by CPAF, and high flow shunting through the fistula has been suggested to contribute to progressive dilation or aneurysm formation.^10,11)^ Another proposed mechanism involves a pressure gradient between the right and left coronary arteries, which has been reported to promote dilation or aneurysm formation of VAR^5)^; however, no significant stenosis or occlusion was observed in either artery in the present case. Notably, this is, to our knowledge, the first report to discuss the mechanisms underlying pathological changes in VAR based on histopathological examination. Histopathological examination of the VAR specimen revealed infiltration of CD8-positive T cells, in addition to atherosclerotic changes. CD8-positive T cells have been reported to contribute to coronary artery inflammation and aneurysm formation in patients with Kawasaki disease,^13)^ whereas their association with coronary artery aneurysms in patients without Kawasaki disease has not yet been demonstrated. In addition, CD8-positive T cells contribute to atherosclerosis, endothelial injury, and plaque destabilization.^14)^ Furthermore, inflammation mediated by CD8-positive T cells activates the production of matrix metalloproteinase, which degrade collagen and elastin, thereby leading to fragility of the vascular wall.^15)^ In the present case, histopathological examination of the resected VAR revealed infiltration of CD8-positive T cells, and examination of the resected coronary artery aneurysm wall demonstrated thinning of the vessel wall and disruption of elastic fibers in the media. Based on these findings, it is suggested that inflammation associated with CD8-positive T cells infiltrating the VAR contributed to vascular wall fragility, including thinning of the vessel wall and disruption of elastic fibers, ultimately leading to dilation and aneurysm formation.

There is no clear evidence guiding the management of VAR, and treatment decisions should be based on patient-specific symptoms and clinical features. VAR itself is typically asymptomatic and is unlikely to require therapeutic intervention, whereas invasive treatment may be considered in symptomatic cases, particularly when complicated by giant CAA (≥20 mm), CPAF, or infective endocarditis.^7,9–12)^ Although invasive treatment options for VAR include surgical repair and catheter-based intervention, surgery is generally recommended in cases with a large shunt, multiple communications, tortuous or aneurysmal vessels, or giant aneurysms.^6,10–12)^ In the present case, the patient was symptomatic and had multiple CAAs, with a maximum diameter of 27 mm, concomitant with a CPAF; therefore, invasive treatment was indicated. Given the marked tortuosity of the VAR, catheter-based intervention was considered technically challenging, and surgery was selected. Alternatively, medical treatment, including antiplatelet agents, calcium channel blockers, β-blockers, and nitrates, has also been reported to alleviate symptoms.^8,12)^ In cases in which invasive treatment is not indicated, it is associated with an unacceptably high risk, is declined by the patient, and conservative management may be the appropriate alternative; however, given the potential for progression of VAR-associated coronary aneurysms,^9)^ careful follow-up with serial CT is warranted.

## CONCLUSIONS

We report a rare case of VAR complicated by multiple CAAs and a CPAF, and provide an important histopathological discussion on the mechanisms underlying pathological changes in VAR. Our findings suggest that inflammation associated with CD8-positive T cells may contribute to vascular wall fragility in VAR.

## DECLARATIONS

### Availability of data and materials

The data are available from the corresponding author upon reasonable request.
